# Lung Nodule Detection *via* Deep Reinforcement Learning

**DOI:** 10.3389/fonc.2018.00108

**Published:** 2018-04-16

**Authors:** Issa Ali, Gregory R. Hart, Gowthaman Gunabushanam, Ying Liang, Wazir Muhammad, Bradley Nartowt, Michael Kane, Xiaomei Ma, Jun Deng

**Affiliations:** ^1^Department of Therapeutic Radiology, School of Medicine, Yale University, New Haven, CT, United States; ^2^Department of Chronic Disease Epidemiology, School of Public Health, Yale University, New Haven, CT, United States; ^3^Department of Radiology and Biomedical Imaging, School of Medicine, Yale University, New Haven, CT, United States; ^4^Department of Biostatistics, School of Public Health, Yale University, New Haven, CT, United States

**Keywords:** lung cancer, computed tomography, lung nodules, computer-aided detection, reinforcement learning

## Abstract

Lung cancer is the most common cause of cancer-related death globally. As a preventive measure, the United States Preventive Services Task Force (USPSTF) recommends annual screening of high risk individuals with low-dose computed tomography (CT). The resulting volume of CT scans from millions of people will pose a significant challenge for radiologists to interpret. To fill this gap, computer-aided detection (CAD) algorithms may prove to be the most promising solution. A crucial first step in the analysis of lung cancer screening results using CAD is the detection of pulmonary nodules, which may represent early-stage lung cancer. The objective of this work is to develop and validate a reinforcement learning model based on deep artificial neural networks for early detection of lung nodules in thoracic CT images. Inspired by the AlphaGo system, our deep learning algorithm takes a raw CT image as input and views it as a collection of states, and output a classification of whether a nodule is present or not. The dataset used to train our model is the LIDC/IDRI database hosted by the lung nodule analysis (LUNA) challenge. In total, there are 888 CT scans with annotations based on agreement from at least three out of four radiologists. As a result, there are 590 individuals having one or more nodules, and 298 having none. Our training results yielded an overall accuracy of 99.1% [sensitivity 99.2%, specificity 99.1%, positive predictive value (PPV) 99.1%, negative predictive value (NPV) 99.2%]. In our test, the results yielded an overall accuracy of 64.4% (sensitivity 58.9%, specificity 55.3%, PPV 54.2%, and NPV 60.0%). These early results show promise in solving the major issue of false positives in CT screening of lung nodules, and may help to save unnecessary follow-up tests and expenditures.

## Introduction

Computed tomography (CT) is an imaging procedure that utilizes X-rays to create detailed images of internal body structures. Presently, CT imaging is the most preferred method to screen the early-stage lung cancers in at-risk groups (1). Globally, lung cancer is the leading cause of cancer-related death (2). In the United States, lung cancer strikes 225,000 people every year and accounts for $12 billion in healthcare costs (3). Early detection is critical to give patients the best chance of survival and recovery.

Screening high risk individuals with low-dose CT scans has been shown to reduce mortality (4). However, there is significant inter-observer variability in interpreting screenings as well as a large number of false positives which increase the cost and reduce the effectiveness of screening programs. Given the high incidence of lung cancer, optimizing screening by reducing false positives and false negatives has significant public health impact by limiting unnecessary biopsies, radiation exposure, and other secondary costs of screening (5).

Several studies have shown that imaging can predict lung nodule presence to a high degree (6). Clinically, detecting lung nodules is a vital first step in the analysis of lung cancer screening results—the nodules may or may not represent early-stage lung cancer. Numerous computer-aided detection (CAD) methods have been proposed for this task. The majority, if not all, utilize classical machine learning approaches such as supervised/unsupervised methods (7). The goal of this work is to adopt for the first time a reinforcement learning (RL) algorithm for lung nodule detection. Developed by Google DeepMind, RL is a cutting-edge machine learning approach which has improved upon numerous CAD systems and helped to beat the best human players in the game of Go, one of the most complex games humans ever invented (8). Here, we apply RL to the lung nodule analysis (LUNA) dataset and analyze the performance of the RL model in detecting lung nodules from thoracic CT images.

## Materials and Methods

### Lung Nodule Data

**Figure 1:**
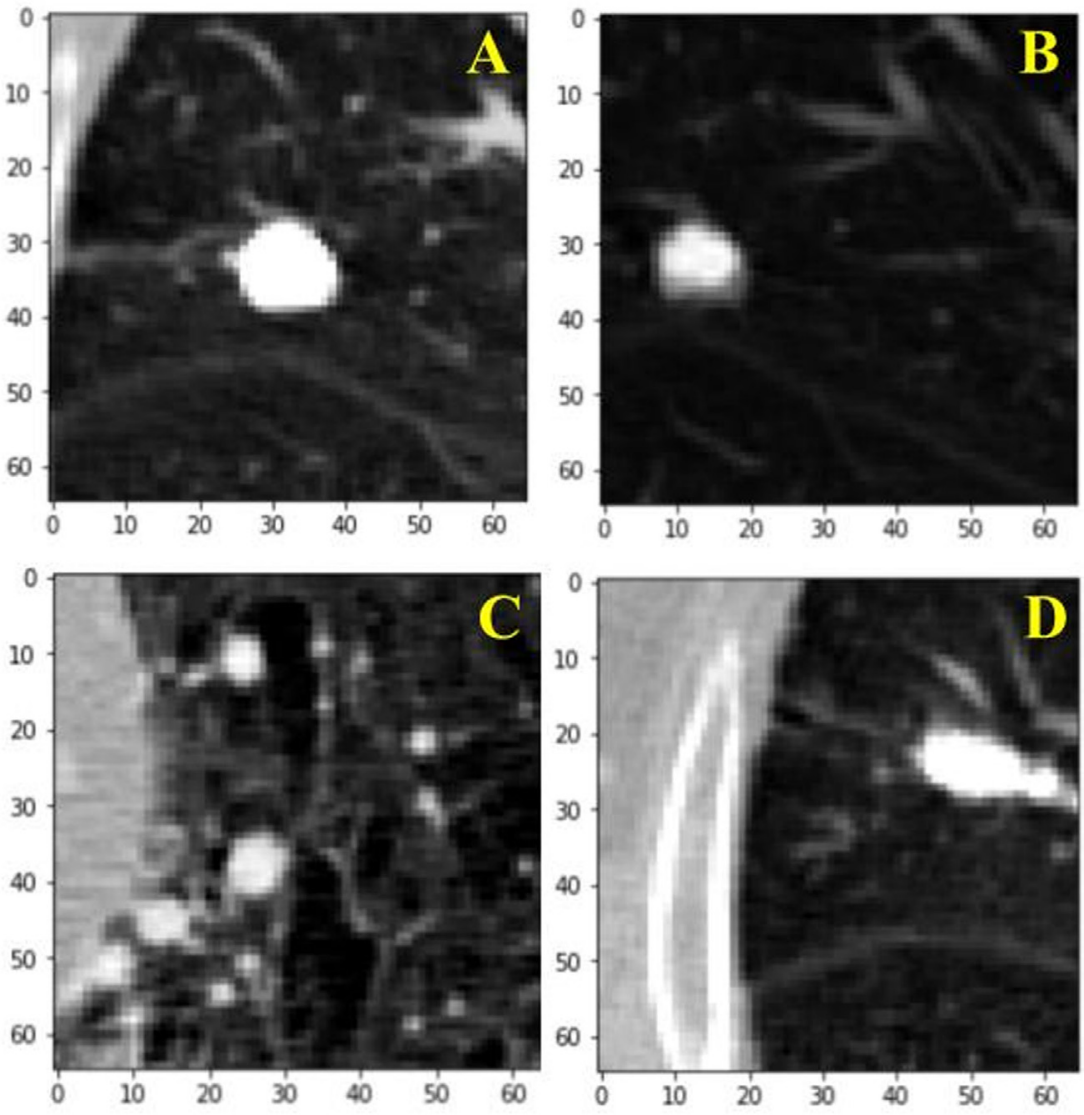
Visual illustration of a sample nodule and non-nodule structure in the lung nodule analysis dataset. Frame **(A)** is a nodule. Frames **(B–D)** are non-nodules.

For the training of our algorithm, we utilize the LUNA dataset, which curates CT images from publicly available LIDC/IDRI database. In total, there are 888 CT scans included. The database also contains annotations collected in two phases with four experienced radiologists. Each radiologist marked lesions they identified as non-nodule (<3 mm) and nodule (≥3 mm) and the annotation process has been described previously (9). The reference standard consists of all nodule ≥3 mm accepted by at least three out of four radiologists. Annotations that are not included in the reference standard (non-nodules, nodules <3 mm, and nodules annotated by only one or two radiologists) are referred to as irrelevant findings (9). A key benefit of this dataset is the inclusion of voxel coordinates in the annotation of nodules, which proves immensely useful when using a RL approach, described in the next section. Figure 1 illustrates examples of nodule and non-nodules from a single CT scan.

### Data Normalization

To balance the intensity values and reduce the effects of artifacts and different contrast values between CT images, we normalize our dataset. The *Z* score for each image is calculated by subtracting the mean pixel intensity of all our CT images, μ, from each image, *X*, and dividing it by σ, the SD of all images’ pixel intensities. This step is helpful when inputting information into a neural network because it fine-tunes the input information fed into a convolution algorithm (10).

(1) $$Z=\frac{X-\mu }{\sigma }$$

### Reinforcement Learning

**Figure 2:**
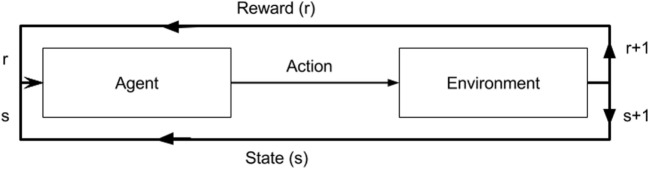
A diagram of a reinforcement model. An agent in a given state (s) and reward (r) completes an action in environment. This results in change of environment and either an increase/decrease in reward as a result of that action.

Reinforcement learning is the science of mapping situations to actions (11). It is a type of machine learning that bridges the well-established classical approaches of supervised and unsupervised learning, where target values are known and unknown, respectively. RL differs in that it seeks to model data without any labels, but rather with incremental feedback. Its recent popularity stems from its ability to develop novel solution schemas, even outperforming humans in certain domains, because it learns to solve a task by itself (12). Essentially, it is a way of programming agents by either a reward or a punishment without the need to specify how a task is to be achieved. A simple RL model is shown in Figure 2 illustrating how an agent’s actions in a given environment affect its resulting reward and state. In its infancy, RL was inspired by behavioral psychology, where agents (i.e., rodent) learned tasks by being given a reward for a correct action taken in a given state. This mechanism ultimately creates a feedback loop. Whether the agent, in our case a neural network model, navigates a maze, plays a game of ping pong, or detects lung nodules, the approach is the same.

A basic reinforcement algorithm is modeled after a Markov decision process. For a set number of states, there are a given number of possible actions, and a range of possible rewards (13). To help optimize an agent’s actions a *Q*-learning algorithm is used (14).

(2) $$Q\left(s_{t},a_{t}\right)=Q\left(s_{t},a_{t}\right)+l*\left[r_{t+1}+\text{max}Q\left(s_{t+1},a_{t}\right)-Q\left(s_{t},a_{t}\right)\right]$$

How a model knows the potential rewards from taking a certain action comes from experience play. That is, it stores numerous combinations of state to state transitions (*s*→*s*^+1^), with the corresponding action, *a*, taken by the model and the resultant reward, *r*: denoted as (*s, a, r, s*^+1^). For instance, in a game environment, the best action to take would be the action that leads to the greatest future rewards (i.e., winning the game), even though the most immediate action may not be rewarding in the short term. As shown in Eq. 2, the expected future rewards are approximated by multiplying the discount rate, λ, by the value of the action that would return the largest future reward based on all possible actions, max*Q*(*s_t_*_+1_, *a_t_*). For a given action, what is learned is the reward for that action, *r_t_*_+1_, plus the largest future reward expected less current action value, *Q*(*s_t_, a_t_*). This is learned at a rate, *r*, the extent to which the algorithm overrides old information, and it is valued between 0 and 1. To learn which series of actions result in the greatest number of future rewards, RL algorithms depend on both greedy and exploratory search. The two methods allow a model to explore all possible ways to accomplish a task, and select the most efficient rewarding (12).

Using the RL approach to tackle the lung nodule task requires one main adaptation, which is how we define a state. In a typical RL task, a state would refer to a snapshot of everything in an environment at a certain time. However, with lung CT images, which are a collection of axial lung scans, we define a *state* as every 10 stacked axial images. Hence, our environment is very deterministic. That is, any *action* taken in a lung CT image state would lead to the succeeding 10 scans, from top to bottom. Whereas in a conventional task, such as playing a game, depending on the action there is more than one succeeding state possible. This key difference adapts our reward function to act solely as a reward function and evaluate a state on whether it immediately has a reward or not, instead of incorporating a value function which factors the total reward our agent can expect from a given state in the distant future. This makes logical sense given that there is only one possible distant future in our radiographic image environment, whereas in a game environment there is more than one possible distant future. As such, rewards are 1 and 0, depending on whether a classification is correct or incorrect, respectively, for the immediate state at hand only. Thus, the memory replay used to train our model, excludes the succeeding state, and only captures current state, action, and reward.

### Convolutional Neural Networks (CNNs)

Learning to control agents directly from high dimensional sensory inputs (i.e., vision and speech) is a significant challenge in RL (11). A key component of our RL model is a CNN. It helps our model make sense of the very high dimensional CT images that we insert into our model. A standard slice has a width and length of 512 × 512. With our input of 10 slices for every state, this amounts to approximately 2,621,440 pixels. A CNN is able to contend with this because it creates a hierarchical representation of high dimensional data such as an image (10).

Unlike a regular neural network, the layers of a CNN have neurons arranged in three dimensions (width, height, and depth) and respond to a receptive field, a small region of the input image, as opposed to a fully connected layer which responds to all the neurons. For a given neuron, it learns to detect features from a local region, which facilitates the capturing of local structures while preserving the topology of the image. The final output layer reduces the image into a vector of class scores. A CNN deep learning system is composed of five layers: an input layer, a convolutional layer, an activation layer, a pooling layer, and a fully connected layer. With most CNN architectures having more than one of each layer, they are thus referred to as “deep” learning (10). The function of each layer is described further below.

#### Input Layer

**Figure 3:**
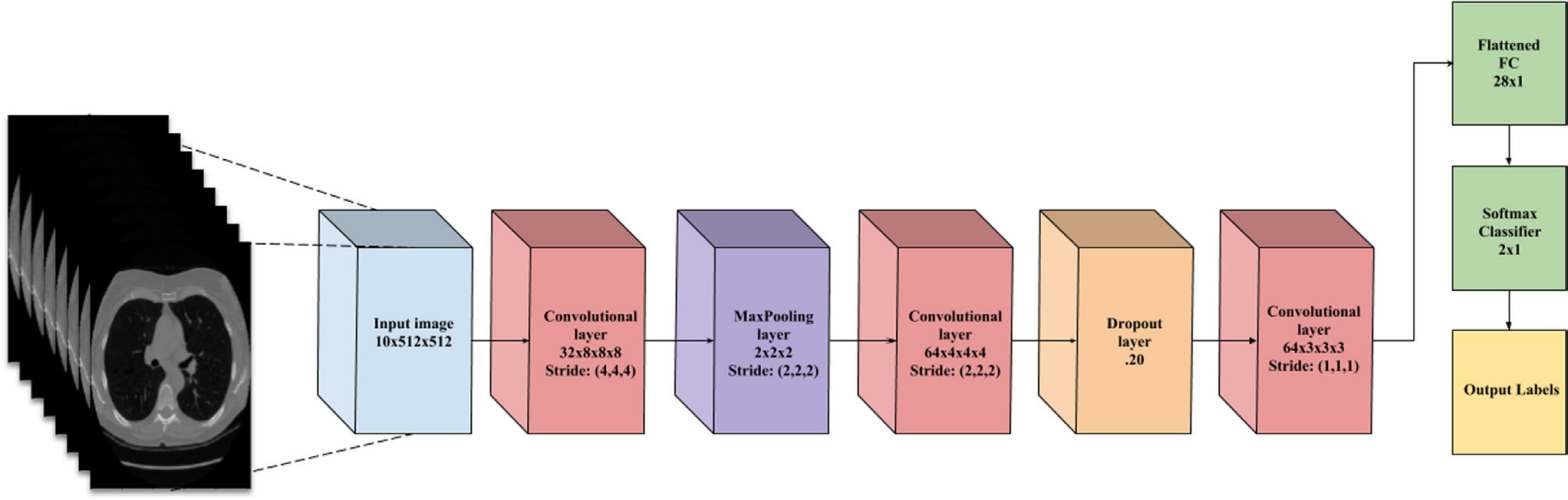
A flowchart of the convolutional neural network architecture. Blue box is the input image. Red boxes are convolutional layers with rectified linear unit activation. Purple box is the max pooling layer. Cyan box is the dropout layer. Green box is the fully connected layer and softmax binary classifier. Yellow is the output of the network.

This layer holds the raw pixels values of the input image (colored blue in Figure 3).

#### Convolutional Layer

This layer visualized by the red boxes in Figure 3 is composed of several feature maps along the depth dimension, each corresponding to a different convolution filter. All neurons with the same spatial dimension are connected to the same receptive field of the input image. This facilitates capturing a wide variety of imaging features. The depth of the layer, meaning the number of convolution filters, represents the number of features that can be extracted from each input receptive field. Each neuron in a feature map shares exactly the same weights, which define the convolution filter. This allows reducing the number of weights, and thus increasing the generalizability of the architecture (10).

#### Activation Layer

Often seen as one with the convolutional layer, as in Figure 3, the activation layer applies a threshold function to the output of each neuron in the previous layer. In our network, we use a rectified linear unit (RELU) activation, where RELU(*x*) = max(0, *x*), meaning it fires the real value of the output and thresholds at zero. It simply replaces the negative values with “0.”

#### Pooling Layer

Typically placed after an activation layer, this layer down-samples along spatial dimensions. Shown by the purple box in Figure 3, it selects the invariant imaging features by reducing the spatial dimension of the convolution layer. The most commonly used is max pooling, which selects the maximum value of four of its inputs as the output, thus preserving the most prominent filter responses.

#### Fully Connected Layer

Shown as green in Figure 3, this layer connects all neurons in the previous layer with a weight for each connection. As the output layer, each output nodes represents the “score” for each class.

To facilitate the learning of complex relationships, multiple convolutional-pooling layers are combined to form a deep architecture of nonlinear transformations, helping to create a hierarchical representation of an image. This allows learning complex features with predictive power for image classification tasks (10). As illustrated in Figure 3, we use 3D CNN given that nodules are spherical in shape, and can best be captured with 3D convolutions.

### Data Augmentation

Overfitting is a result of network parameters greatly outnumbering the number of features in the input images. Given the network size and the number of features available from the CT images, our model tended to overfit, hence the need to increase the number of CT images. To counter this overfitting, we used standard deep neural network methods, such as artificially augmenting the dataset using label-preserving transformations (15). The data augmentation consists of applying various image translations, such as rotations, horizontal and vertical flipping, and inversions. We apply a random combination of these transformations on each image, thus creating nominally “new” images. This multiplies the dataset by many folds and helps in reducing overfitting (10).

## Implementation and Experiments

### Implementation

Our python code uses the Keras package (16) and makes use of the Theano Library. Keras can leverage graphical processing units to accelerate the deep learning algorithms. We trained our CNN architecture on an NVIDIA Quadro M6000 GPU card. Training time was approximately 2 h.

### Experimentation

We utilize the entire LUNA dataset (*n* = 888 patients), with 70% in training our model and 30% in test. In the training set, we balance our dataset for nodule states and non-nodule states. As shown in Table 1, for any sampling of states selected, approximately 5% are nodule states. Early on, the imbalance caused our model to bias significantly toward detecting non-nodule states given that those are the majority of states. The balanced dataset contains a total of 2,296 states, with 1,148 nodule states and 1,148 non-nodule states. It was created by retrieving nodule states from every patient with a nodule and random non-nodule states from all patients. For every epoch during the training, 20% of the training set is separated for cross-validation.

**Table 1:** The number of patients and nodules they carry for nodule versus non-nodule groups.

	# of patients	# of states	# of nodules
Nodules	590	15,616	1,148
Non-nodules	298	7,107	0

For our model, the sensitivity, specificity, accuracy, positive predictive value (PPV), and negative predictive value (NPV) were computed as follows:

Sensitivity or true positive rate:
  $$\text{TPR=}\frac{\text{TP}}{\text{TP+FN}}$$
Specificity or true negative rate:
  $$\text{TNR=}\frac{\text{TN}}{\text{TN+FP}}$$
Accuracy:
  $$\frac{\text{TP+TN}}{\text{TP+FN+TN+FP}}$$
PPV:
  $$\text{PPV=}\frac{\text{TP}}{\text{TP+FP}}$$
NPV:
  $$\text{NPV=}\frac{\text{TN}}{\text{TN+FN}}$$

where TP, FP, TN, and FN stand for true positive, false positive, true negative, and false negative, respectively.

## Results

**Figure 4:**
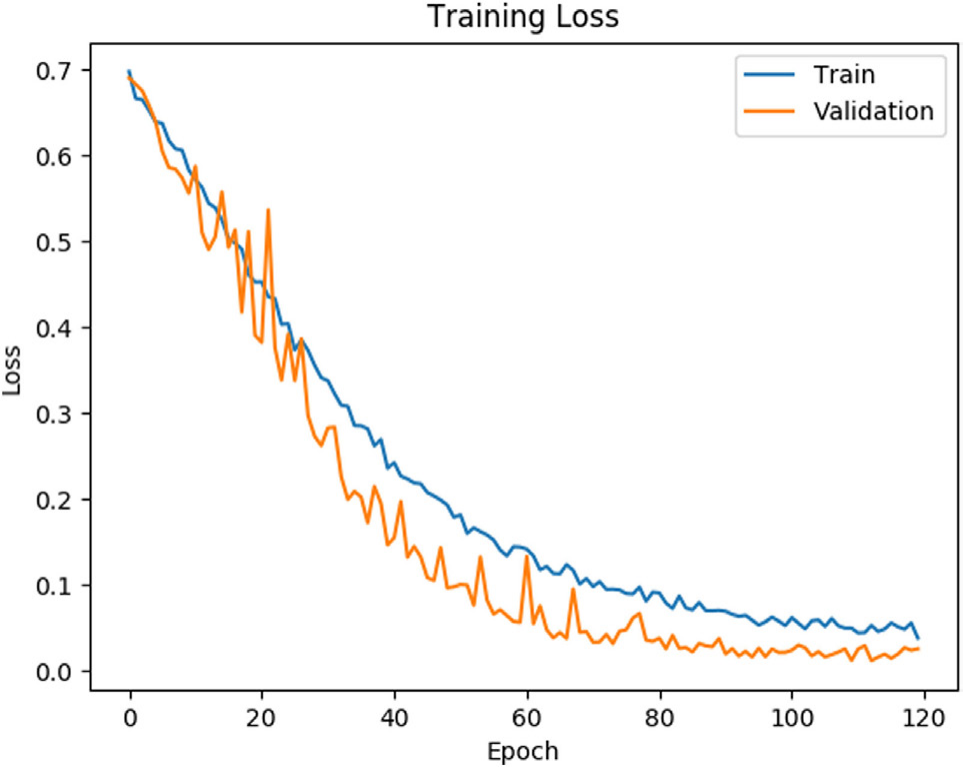
Training and validation loss is shown on the training data for 120 epochs. Blue line corresponds to training loss and orange line corresponds to validation loss.

**Figure 5:**
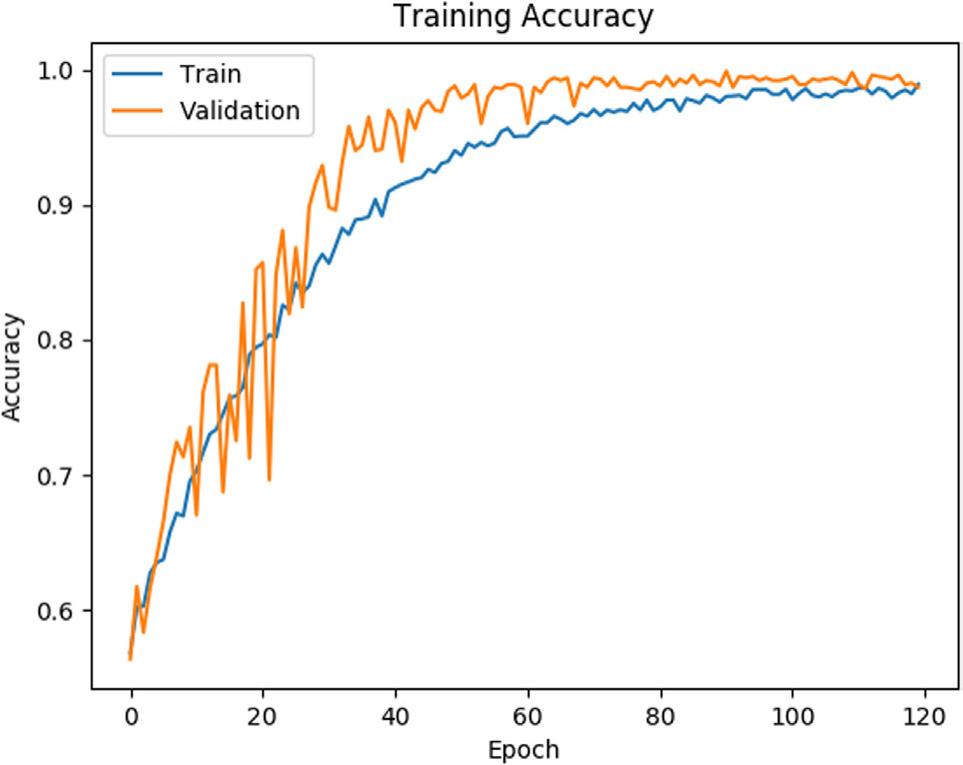
Training and validation accuracy is shown for the training data for 120 epochs. Blue line corresponds to training accuracy and orange line corresponds to validation accuracy.

As shown in Figures 4 and 5, for both loss and accuracy we observed a steady improvement. In Figure 4, showing the loss value over time, or epochs, there is a steady decline to approximately zero. A similar pattern holds with accuracy, in Figure 5, but with the steady increase to a value of one, meaning perfect score. Both graphs were generated from training on 70% of the dataset (1,607 states) and cross-validating on 20% of that (321 states). As observed in both graphs, the model is “learning”, however there still remains considerable volatility as shown by the validation curves.

The conclusive results from the training and testing for our model is detailed in Table 2 The test sample size was 30% of the dataset (668 states).

**Table 2:** The sensitivity, specificity, accuracy, positive predictive value (PPV), and negative predictive value (NPV) PPV results are listed for our reinforcement model from training and from testing.

	Accuracy	Sensitivity	Specificity	PPV	NPV
Training	99.1%	99.2%	99.1%	99.1%	99.2%
Test	64.4%	58.9%	55.3%	54.2.6%	60.0%

**Figure 6:**
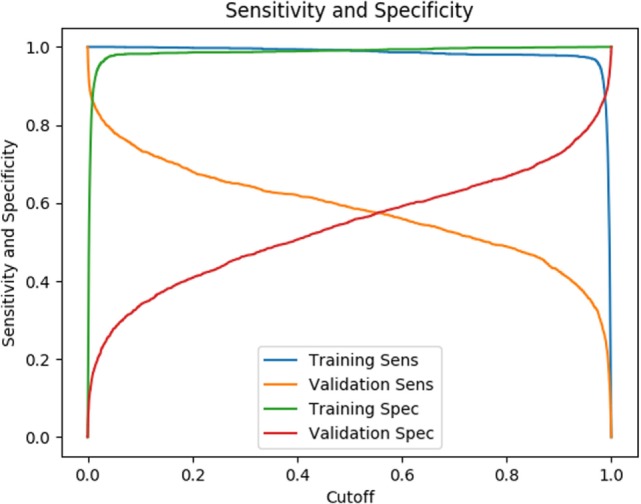
Sensitivity and specificity as a function of cutoff, the likelihood a state has a nodule.

The testing results listed in Table 2 are based on a cutoff value of 0.5. Given our model is a binary classifier, this means that for any state that it predicts, the likelihood of nodule is at least 0.5. Figure 6 illustrates how the sensitivity and specificity vary as functions of cutoff values for both training and testing results.

## Discussion

In this study, we present a robust non-invasive method to predict the presence of lung nodules, a common precursor to lung cancer, from lung CT scans using a RL method. A major advantage of this approach is that it allows to develop novel and unpredictable solutions to complex problems. From the results of our training using the LUNA dataset, we were able to achieve superb sensitivity, specificity, accuracy, PPV, and NPV (all greater than 99%). While the metrics for the testing dataset were lower, they were consistent. In both data size and number of trials, we achieved similar results. This consistency suggests that our research approach of using RL with non-pre-processed data is reproducible. Moreover, given the nature of RL, the model will only continue to improve with time and more data.

The way in which RL algorithms continue to improve depends not only on the quality of the dataset, but also more importantly its size. In the training of the AlphaGo, it was trained on master-level human players, instead of picking up the best strategies to win from scratch (8). In addition, the RL algorithm learned through more than 30 million human-on-human games. Factoring in hardware, AlphaGo required $25 million in computer hardware (17), it was trained on master-level human players (8).

Although the tasks of playing a game of Go is very different from detecting lung nodules, an inference we can draw is that reinforcement learning algorithms, such as AlphaGo, require substantial data to train. Given the original dataset’s small size, there is an inherent difficulty in capturing the huge variability and structural differences in the lung volumes of human beings. With only 888 CT scans and approximately 1,148 nodule states in our dataset, with 70% of that being used for training, the lesson we have learned is that our model needs a significant amount of more data. This is evidenced by the tremendous amount of data and hardware needed to train AlphaGo to reach super human performance.

It is worth noting that AlphaGo’s performance is based on how well it performed against human players. Similarly, our model performance is based on how well it performed against at least three radiologists in detecting lung nodules. As described by Armato et al. (9) how a given lesion was classified as a nodule was determined by a consensus of at least three of the four radiologists. A significant variability is observed when comparing the number of lesions classified as a nodule by one radiologist versus at least three radiologists. For the lesions identified in all the scans, 928 lesions were classified as nodules ≥3 mm by all four radiologists and 2,669 lesions were classified as a nodule ≥3 mm by at least one radiologist. This means for nodules ≥3 mm, the false discovery rate for a given individual radiologist is 65.2% (9). In contrast, despite the overfitting, our model classification yields a false discovery rate of 44.7% on the validation dataset, which is an improvement compared to an individual radiologist.

Given the very high training results, the question of overfitting arises. With a small dataset, the underlying probability distribution of lung nodules is not sufficient to create a fully generalizable model, especially given it is based on RL. As with most parametric tests, a fundamental assumption of samples is that they adequately capture the variance of the population they represent. With small datasets, depending on the variable, a random subset of the data may not adequately capture the variance of the overall dataset. With the LUNA dataset, this is particularly an issue given the fact that it is very high dimensional and our model requires significantly more data to capture the true variance of its countless variables. Most CT image datasets comprise of thousands of images, as compared to the millions of games in AlphaGo, and thus the comparison is not quite the same. We employed dropout and data augmentation to increase the generalizability of our model in response to the overfitting. Together these two approaches have minimally dampened the effect. An alternate approach we also experimented with was to reduce the network size, however, this approach resulted in significant volatility in the training and validation results. Regardless of the overfitting, the performance on the validation data set indicates that our model achieves enough generalization to compete with a human radiologist and could serve as a second reader.

A strength of our research approach is the lack of pre-processing. It is known that medical imaging, including CT images, can be very heterogeneous. From the number of image slices, scanning machine used, and scanning parameters used, the image data for each patient is very disparate. A significant negative byproduct of this heterogeneity is the astronomical number of insignificant features generated that are unrelated to one’s outcome of interest, such as the presence of lung nodules. For a machine learning algorithm to contend with this either the data size has to exponentially increase or many of the insignificant features have to be pre-processed out by filtering for only the relevant features. The former option of increasing the dataset is impractical, as the LUNA dataset is already one of the largest and most comprehensive image datasets. Hence, most, if not all, approaches in the current literature on CAD systems for lung nodule detection take the second option of pre-processing. From using various filters, masks, and general pre-processing tools, these methods heavily curate and alter the raw medical image data. As a result, this can create an infinite number of variations of the original dataset, and such a subjective practice makes it very difficult to reproduce any of the experimental results. We choose to use data without pre-processing to ensure that our results are reproducible.

Our work highlights the promise of using RL for lung nodule detection. There are several practical applications of this model, one of which is to serve as a second opinion or learning system for radiologists and trainees in identifying lung nodules. A strong appeal of using a RL approach is that the model is always in a learning state. With every new patient, the model expands its learning by factoring in the new information and building upon its probabilistic memory of historical information from previous patients. This phenomenon is what allowed the artificial intelligence model AlphaGo to keep improving after each match, eventually beating each player after several matches, including the reigning world champion. Likewise, we expect that our model will continue to improve as it observes more and more cases.

## Author Contributions

IA and GH: carried out primary experiments of project. GG, MK, and XM: provided guidance on methodology and overall project. YL, WM, and BN: provided lab and technical support. JD: generated research ideas, provided guidance on methodology and overall project, and reviewed manuscript.

## Disclaimer

The content is solely the responsibility of the authors and does not necessarily represent the official views of the National Institutes of Health.

## Conflict of Interest Statement

The authors declare that the research was conducted in the absence of any commercial or financial relationships that could be construed as a potential conflict of interest.
